# Classification of Sleep Apnea Severity by Electrocardiogram Monitoring Using a Novel Wearable Device

**DOI:** 10.3390/s20010286

**Published:** 2020-01-04

**Authors:** Florent Baty, Maximilian Boesch, Sandra Widmer, Simon Annaheim, Piero Fontana, Martin Camenzind, René M. Rossi, Otto D. Schoch, Martin H. Brutsche

**Affiliations:** 1 Cantonal Hospital St. Gallen, Lung Center, Rorschacherstrasse 95, 9007 St. Gallen, Switzerland; maximilian.boesch@kssg.ch (M.B.); sandra.widmer@kssg.ch (S.W.); otto.schoch@kssg.ch (O.D.S.); martin.brutsche@kssg.ch (M.H.B.); 2 Empa, Laboratory for Biomimetic Membranes and Textiles, Lerchenfeldstrasse 5, 9014 St. Gallen, Switzerland; simon.annaheim@empa.ch (S.A.); piero.fontana@empa.ch (P.F.); martin.camenzind@empa.ch (M.C.); rene.rossi@empa.ch (R.M.R.)

**Keywords:** sleep apnea, classification algorithms, ECG signal, wearable acquisition device, heart rate variability analysis, support vector machine

## Abstract

Sleep apnea (SA) is a prevalent disorder diagnosed by polysomnography (PSG) based on the number of apnea–hypopnea events per hour of sleep (apnea–hypopnea index, AHI). PSG is expensive and technically complex; therefore, its use is rather limited to the initial diagnostic phase and simpler devices are required for long-term follow-up. The validity of single-parameter wearable devices for the assessment of sleep apnea severity is still debated. In this context, a wearable electrocardiogram (ECG) acquisition system (ECG belt) was developed and its suitability for the classification of sleep apnea severity was investigated using heart rate variability analysis with or without data pre-filtering. Several classification algorithms were compared and support vector machine was preferred due to its simplicity and overall performance. Whole-night ECG signals from 241 patients with a suspicion of sleep apnea were recorded using both the ECG belt and patched ECG during PSG recordings. 65% of patients had an obstructive sleep apnea and the median AHI was 21 [IQR: 7–40] $h^{-1}$. The classification accuracy obtained from the ECG belt (accuracy: 72%, sensitivity: 70%, specificity: 74%) was comparable to the patched ECG (accuracy: 74%, sensitivity: 88%, specificity: 61%). The highest classification accuracy was obtained for the discrimination between individuals with no or mild SA vs. moderate to severe SA. In conclusion, the ECG belt provided signals comparable to patched ECG and could be used for the assessment of sleep apnea severity, especially during follow-up.

## 1. Introduction

Sleep apnea is a common disorder characterized by reduction or cessation of airflow to the lungs caused by obstructive or central events. It is a highly prevalent disease with recent data from the United States and Europe indicating that 14–49% of middle-aged men have clinically relevant sleep apnea [1,2,3]. Sleep apnea syndrome (SAS) is associated with arterial hypertension [4,5] and contributes to the development of overt cerebro- and cardiovascular comorbidities [6,7]. The number of consultations for the diagnosis of SAS have increased and resources are scarce [8]. SAS is diagnosed by polysomnography (PSG) based on the number of apnea–hypopnea events per hour of sleep (apnea–hypopnea index, AHI) [9]. This laboratory-based method is the gold standard for SAS detection and characterization. However, PSG is considered relatively expensive, technically complex and possibly disruptive to sleep [10]. Inter-night variability in the severity of SAS has been reported in the literature [11]. Therefore, patient monitoring during consecutive nights is desirable in both screening and follow-up settings. For multiple reasons, multi-night investigations cannot be done using standard PSG.

Research in the field of sleep medicine has been considered in order to create simpler and cost-effective novel devices mainly for follow-up investigations of patients in order to confirm treatment strategy and evaluate treatment efficacy. In addition, the development of novel portable devices enables patient monitoring in an unattended home setting. To achieve the minimum required data quality, guidelines on portable monitoring are already available [12]. The guidelines include the recommendations that portable monitoring may be used in populations with high pretest probability of moderate to severe SAS which is met in case of follow-up investigations.

Various portable sleep-monitoring devices have been developed and reported in the scientific literature [13,14,15,16,17,18]. With the development of computer-based data analysis, new approaches for the assessment of sleep apnea severity have been reported. Even though guidelines generally recommend the use of multi-sensors approaches in selected patient populations [12], several algorithms have been developed for the detection of sleep apnea from electrocardiogram (ECG) recordings alone [19,20,21,22]. Patients suffering from SAS typically show cyclic variations in the heart rate which translate into cyclic variations in ECG amplitude or morphology. Interval between heartbeats (RR intervals) can be extracted from ECG signals. Depending on signal quality, data pre-processing consisting of filtering out artefacts from the RR time series are recommended. The resulting RR intervals are then analyzed using heart rate variability (HRV). Typically, HRV measures are divided into two broad categories: time-domain measures and frequency-domain measures [23]. These spectral analyses derived from whole-night ECG signals provide a series of features that can be used to classify patients with SAS [24]. Therefore, these algorithms typically include a first step where features are extracted from the raw signal and these features are then used as an input for the generation of a classification model.

For the acquisition of ECG signals, mainly gel electrodes are used. However, gel electrodes are unsuitable for long-term use in an unattended home monitoring setting, because multi-night measurements require the intervention of healthcare personal and repeated attachments can lead to skin irritations. In this context, a novel wearable ECG acquisition system has been developed [25]. The key components of the portable system are porous textile ECG electrodes and a humidification unit enabling continuous multi-night monitoring of ECG signals. As the system is designed as a chest belt, it can be easily applied by the patients themselves in an unattended home setting. In a first study, the validity of this ECG acquisition system was evaluated in patients with SAS by comparing the in-laboratory ECG values (RR intervals) to the ones obtained with patched ECG during PSG. Data were processed to remove artefacts induced by movements or any other source and showed high levels of agreement between ECG measurements acquired from PSG and the wearable acquisition system [26]. Furthermore, the wearable acquisition system was deemed appropriate for unattended sleep apnea monitoring in a home setting [27].

Therefore, the aim of the current study was to evaluate the value of overnight ECG recordings to assess sleep apnea severity using a portable ECG belt in a population of patients with a suspicion of SAS. A direct comparison with the data obtained from patched ECG during PSG was performed and the impact of data pre-processing (filtering of RR intervals) were investigated. Finally the predictive performance of ECG with regard to oxygen desaturation and daytime sleepiness will also be investigated.

## 2. Materials and Methods

### 2.1. Patients

In total, 245 consecutive patients with a suspicion of SAS investigated with a whole-night PSG at the Sleep Laboratory of the Cantonal Hospital St. Gallen were included. Three patients did not complete the study and the patients characteristics from one additional patient were missing. Consequently, in the current study, the data from 241 patients were analyzed. The study was performed in accordance with the Declaration of Helsinki, following the principles of Good Clinical Practice. The study was approved by the local institutional review board (EKSG 15/140) and patients gave written informed consent to participate.

### 2.2. Sleep Apnea Diagnosis Using PSG

The diagnosis of sleep apnea was established in all participating patients using the gold standard PSG. PSG reports were scored by trained physicians with the help of technicians from our sleep laboratory according to standard procedures defined by the American Academy of Sleep Medicine [28].

### 2.3. Wearable ECG-Belt

The ECG belt was previously described in details in previous publications [25,26,27]. It consists of a semi-elastic polyester belt (Unico Swiss Tex GmbH, Alpnachstad, Switzerland) with directly embroidered Ag/Ti-coated PET yarn (Serge Ferrari Tersuisse AG, Emmenbrücke, Switzerland) that forms the electrodes. Furthermore, it contains a water filled wetting pad which delivers approximately 3 g of water per day via evaporation in order to improve signal conduction (Unico Swiss Tex GmbH, Alpnachstad, Switzerland). All materials used for the belt were skin-friendly and non-cytotoxic. Fifteen different ECG belts were used, and the measurement quality was continuously monitored.

### 2.4. Heart Rate Variability Analysis and Features Extraction

ECG signals were simultaneously monitored in every patient from both the wearable ECG belt and the gold standard PSG [26]. Both ECG signals were extracted from EDF files generated by the PSG measurements. Beat-to-beat intervals were automatically generated using the EDFbrowser software (v. 1.62; http://www.teuniz.net/edfbrowser/). A filter of the RR intervals suggested by the Task Force of the European Society of Cardiology and the North American Society of Pacing and Electrophysiology [23] was also tested. Unrealistic short RR intervals for sleep conditions of less than 300 ms or more than 1500 ms were excluded from the analysis. Furthermore, RR intervals differing more than 20% from the median calculated based on the ten preceding and following RR intervals were not considered for further analysis. The impact of this data pre-processing was assessed by comparing the ECG signals with or without filtering. HRV analysis was applied to the whole ECG recording, including both intervals with and without apnea/hypoapnea events. Therefore, no prior apnea/hypoapnea event identification was needed. HRV analysis was performed by means of time-domain and frequency-domain analyses. In order to perform such analyses, the width of the window used to analyze short segments of RR time series needs to be specified in advance. A typical window width of 300 s was used. The displacement of the window used for calculating the spectrograms was set to 10 s. For sensitivity purpose another window size of 100 s was used. Power bands of the heart rate signal were calculated using short-time Fourier transform. From the time-domain analysis, the following features were extracted: standard deviation of the NN intervals (SDNN), the proportion of interval differences of successive RR intervals greater than 50 ms (pNN50), the root mean square of successive differences (rMSSD), the inter-quartile range of the RR time series (IRRR), the median of the absolute values of the RR time series (MADRR) and the heart rate variability triangular index (HRVi). Time-domain indices generally provide information on the variability of the autonomous nervous system, mainly dependent on the parasympathetic nervous system. From the frequency-domain analysis, the following features were extracted: ultra low frequency (ULF), very low frequency (VLF), low frequency (LF), high frequency (HF), ratio of low frequency on high frequency (LFHF). Frequency-domain indices typically provide information about rhythmic oscillations of the RR intervals. High frequency indices generally provide indications regarding the activity of the parasympathetic nervous system, whereas the low frequency indices rather reflect the activity of the sympathetic nervous system.

### 2.5. Methodology and Data Treatment

Linear combinations of the extracted HRV features were explored using principal component analysis (PCA). The role played by external explanatory variables including patient baseline characteristics, AHI, oxygen desaturation index (ODI), Epworth sleepiness scale (ESS) and type of apnea was further investigated using a vector fitting procedure which identifies the directions of maximal correlation with the external variables in the PCA space. The significance of the fitted vectors was assessed using permutation of the external explanatory variables [29]. The reported *p*-value was obtained by comparing the squared correlation coefficients ($R^{2}$) of the fitted vectors to the distribution of $R^{2}$ obtained after random permutation (tail probability of the Null distribution of the test statistic). A surface fitting procedure was also applied to PCA in order to further characterize the gradient of AHI, ODI and ESS (response) in relationship to the HRV features (predictors). If the relationship between the response and predictors is linear, the fitted contours should be equally separated values perpendicular to the fitted vectors. In a second step, several classification algorithms were used to assess the classification accuracy of patients’ sleep apnea severity based on AHI, ODI and ESS. A few alternative classifiers including support vector machines (SVM), linear discriminant analysis (LDA), *k*-nearest neighbour (KNN) and orthogonal partial least squares (OPLS) were considered and compared with each other. Several cut-offs based on the quantiles of the response variables (AHI, ODI and ESS) were investigated and the sensitivity/specificity of the HRV-based classifiers was assessed and reported graphically using receiver operating characteristic (ROC) curves. The area under the ROC curves was also provided as an indicator of the performance of the classifiers. All analyses were done using the R statistical software [30], including the extension packages RHRV [31], ade4 [32] and vegan [33].

## 3. Results

### 3.1. Patient Characteristics

Patient characteristics are summarized in Table 1. Seventy six percent of patients were males. The median age was 52 years old and the median body mass index (BMI) was 45 kg/m^2^. Patients’ sleep apnea severity was characterized based on PSG observations by a median AHI of 21 events per hour, a median ODI of 17 desaturations per hour and a median ESS score of 9. Roughly two thirds of patients (65%) had an obstructive sleep apnea. Forty patients (17%) were diagnosed with central or mixed sleep apnea, whereas no apnea events were detected in 44 patients (18%).

### 3.2. Heart Rate Variability Feature Extraction

**Figure 1:**
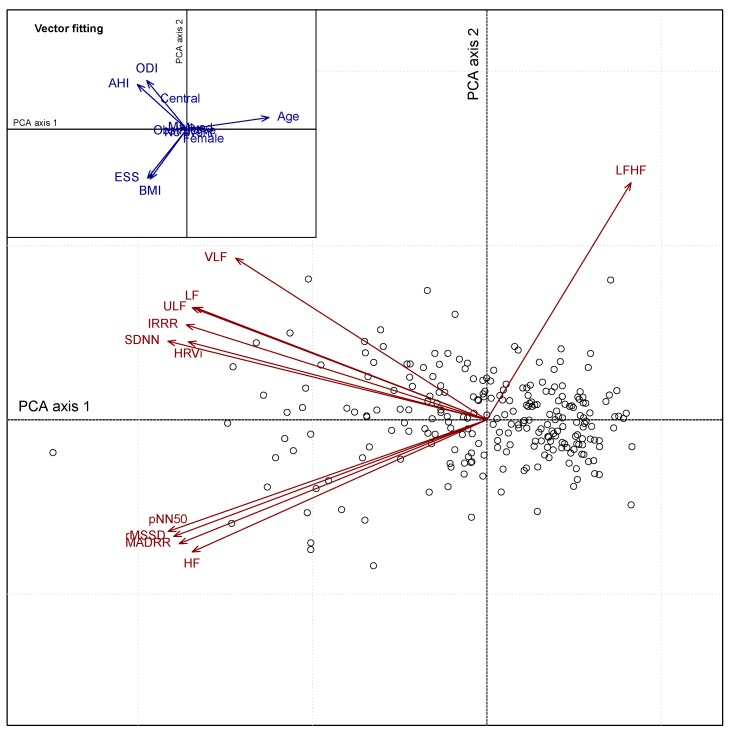
PCA biplot representation of the HRV features (red arrows) and patient information (empty circles) derived from filtered ECG belt data. External explanatory variables including patient baseline characteristics were fitted to the PCA for interpretation purposes and represented by blue arrows oriented towards the direction of maximum correlation (inset on the upper left corner).

HRV analyses from 241 whole night ECG data sets derived from the ECG belt and patched ECG recordings were performed. HRV features were extracted and analyzed in a exploratory manner using PCA. Figure 1 depicts the correlations between HRV features (filtered ECG belt) using PCA. The biplot representation displays both variables (red arrows) and patient information (unique patient number) on the two main PCA axes. The first and second PCA axes explain 69% and 14% of the total variance, respectively. Two clusters of HRV features can be identified from the PCA biplot. A first cluster in the upper left quadrant group together the following correlated features: HRVi, SDNN, IRRR, ULF, LF and VLF. A second cluster in the lower left quadrant regroup the following highly correlated features: HF, MADRR, rMSSD and pNN50. Both clusters contribute to the first axis of the PCA which displays a gradient of apnea severity (from right to left). Independently from these two clusters, the feature LF/HF is oriented towards the upper right quadrant. Using a vector fitting approach (Figure 1, inset on the upper left corner), one can relate the HRV features and associated clusters with various explanatory variables. The first cluster (upper left) is positively associated with both AHI and ODI, reflecting disease severity. The second cluster (lower left) is related to both ESS and BMI. A permutation procedure associated with the vector fitting approach showed an overall significant association between the HRV features and both AHI (p=0.003) and ODI (p=0.018), however no significant association was found with regards to ESS (p=0.871).

### 3.3. Classification of Sleep Apnea

#### 3.3.1. Comparison of Classifiers

**Figure 2:**
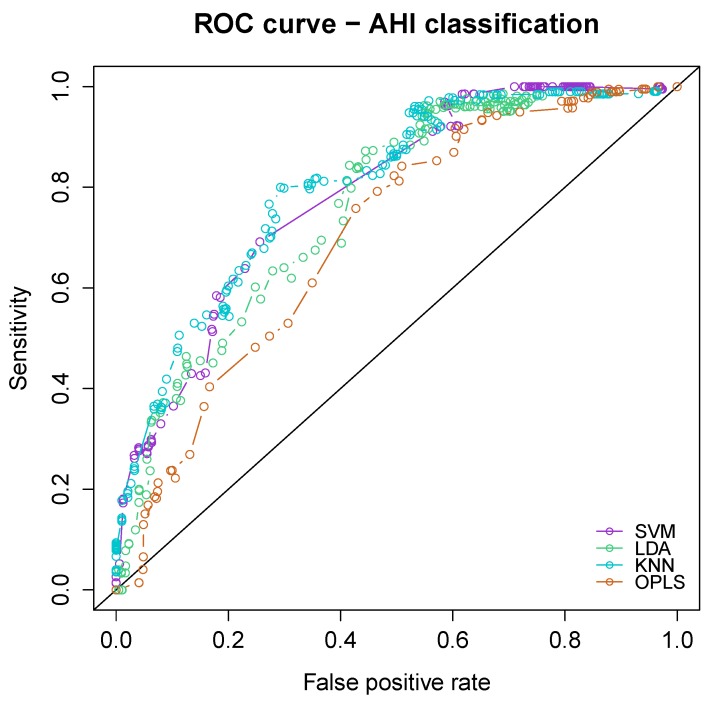
ROC curves displaying the comparison of the classification performance of four different algorithms with respect to sleep apnea severity (apnea–hypopnea index, AHI). The algorithms included support vector machine (SVM), linear discriminant analysis (LDA), k-nearest neighbour (KNN) and orthogonal partial least squares.

The classification performance in terms of sleep apnea severity (AHI) was compared between four alternative classifiers including SVM, LDA, KNN and OPLS. ROC curve analyses are provided in Figure 2. All four algorithms showed similar classification performance with an area under the curve of the four classifiers ranging from 0.702 [95% CI: 0.636 to 0.767] for OPLS up to 0.787 [95% CI: 0.730 to 0.845] for SVM. Support vector machine was eventually preferred due to its simplicity and overall performance. The comparisons of classification accuracy of the ECG belt and patched ECG provided in the next section of the manuscript are derived from SVM only.

#### 3.3.2. Classification of Sleep Apnea Severity, Oxygen Desaturation and Daytime Sleepiness

**Figure 3:**
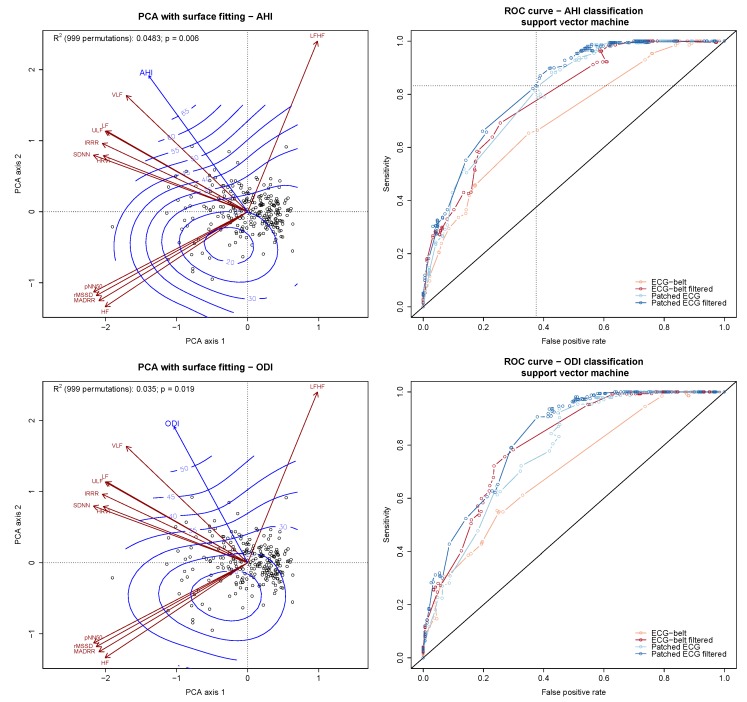

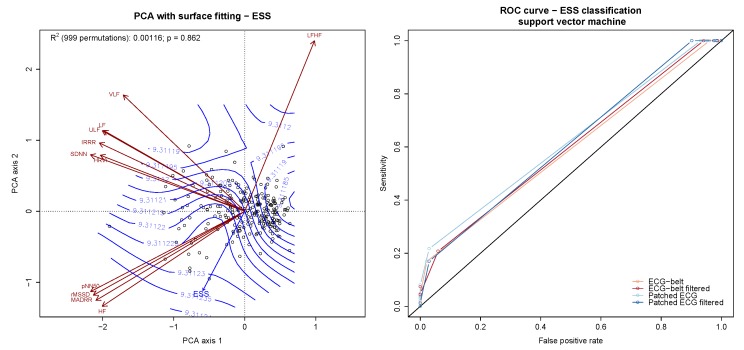
Predictive and diagnostic accuracy of the HRV features with regards to AHI, ODI and ESS. The predictive accuracy of the HRV features is provided by surface fitting applied to PCA (**left panels**). The isoclines (blue lines) superimposed to the PCA biplot display the linear relationship (trend surface) between HRV features and the responses (AHI, ODI, ESS; blue arrows). The diagnostic accuracy is shown by means of ROC curve displaying the sensitivity vs. false positive rate of a support vector machine-based AHI classifier (**right panels**). The curves are derived from ECG data obtained during PSG and ECG belt with or without data pre-filtering. The diagonal line indicates the random guess line. The dotted lines of the upper right panel cross at the optimal classification accuracy for AHI (AHI = 18 $h^{-1}$).

Features extracted from HRV analysis of the ECG belt and patched ECG with and without RR interval filtering were used to further assess the predictive performance of the ECG belt in terms of sleep apnea severity, oxygen desaturation and daytime sleepiness. The prediction accuracy was assessed for the 3 response variables treated either continuously or after dichotomization. The relationship between the HRV features and the continuous response variables was assessed using surface fitting applied to PCA (Figure 3, left panels). The classification accuracy of the dichotomized response variables was assessed by using a SVM-classifier with the HRV features as input. The 3 response variables were dichotomized into high vs. low values by setting cut-off values at different thresholds (percentiles of AHI, ODI and ESS). The performance (sensitivity, specificity and accuracy) of the classifiers was then assessed for each response variable at each cut-off value. The associated curves displaying the sensitivity and false positive rate are shown in Figure 3, right panels.

The relationship between the HRV features and AHI is shown in the upper left panel of Figure 3. A significant linear gradient with equally spaced fitted contours perpendicular to the fitted vector was found ($R^{2}=$ 5%; p=0.003). The diagnostic accuracy of the HRV features with regards to AHI is shown in the ROC curves depicted in Figure 3 (upper right panel). The area under the curves of the unfiltered ECG belt and patched ECG recordings were 0.710 [95% CI: 0.645 to 0.775] and 0.759 [95% CI: 0.698 to 0.820], respectively. The area under the curves of the filtered ECG belt and patched ECG recordings were improved to 0.787 [95% CI: 0.730 to 0.845] and 0.822 [95% CI: 0.769 to 0.876], respectively. The optimal classification accuracy obtained from the filtered ECG derived from patched ECG was 74% (sensitivity: 88%; specificity: 61%). Similar levels of accuracy were achieved by the unfiltered patched ECG (accuracy: 72%, sensitivity: 83%, specificity: 62%) as well as the filtered ECG belt (accuracy: 72%, sensitivity: 70%, specificity: 74%). On the other hand, the unfiltered ECG belt showed significantly worse classification accuracy (accuracy: 65%, sensitivity: 65%, specificity: 65%). The AHI cut-off for which the highest classification accuracy was reached was 18 $h^{-1}$. In this situation, ECG-based classifications proved to be particularly useful for the classification of patients with no or mild SAS versus patients with moderate to severe SAS.

A similar significant relationship between ODI and the HRV features was found (Figure 3, central left panel). A significant linear gradient was characterized ($R^{2}=$ 4%; p=0.018). The classification accuracy between patients with low vs. high ODI was also assessed and showed results mostly in agreement with what was found regarding AHI. An optimal ODI cut-off was 10 $h^{-1}$, with 75% accuracy (sensitivity: 79%; specificity: 71%). The patched ECG (both filtered and unfiltered) gave similar levels of classification accuracy, whereas the unfiltered ECG recording from the unfiltered ECG belt gave significantly worse results.

Poor prediction accuracy was obtained with regards to ESS (Figure 3, lower panels). The relationship between the predictors (HRV features) and response (ESS) was inconsistent (Figure 3, lower left panel) and non-significant ($R^{2}=0.2$%; p=0.880). The classification accuracy of the dichotomized ESS scores was also poor (Figure 3, lower right panel).

## 4. Discussion

Based on a large cohort of patients (n=241), we here assessed the diagnostic and clinical value of whole-night ECG measurements acquired from a wearable ECG belt in comparison with patched ECG signals acquired during gold standard PSG. Using the current algorithm combining feature extraction from HRV analysis and support machine modeling, we achieved a diagnostic accuracy of roughly 75% to discriminate between no/mild vs. moderate/severe sleep apnea. This performance—even in the light of further potential optimization of the classification algorithm—is insufficient in the diagnostic setting or for screening of sleep apnea in unselected individuals. In this situation, the wearable device may benefit from an extended multi-sensor setting. On the other hand, using HRV features derived from the ECG belt, we were able to identify robust and independent indicators of SAS severity in two dimensions—range of apnea/hypopnea events and oxygen desaturation index. Another dimension defined by the LF/HF ratio, often described as an index of imbalance of the autonomic nervous control system [34], was found to be independent from all the other HRV features. Continuous single-channel ECG recordings could, therefore, deliver valuable information complementary to the current clinical follow-up routine for patients already diagnosed with SAS. Indeed, HRV features showed significant performance for the prediction of ODI as reported elsewhere [35]. As has been shown by others [36], our algorithm demonstrated a poor classification performance with regard to daytime sleepiness as measured by the ESS.

In our previous publication, we showed that the ECG measurements from the ECG belt were mostly in agreement with the ones from patched ECG during PSG [26]. Technically, PSG employs single-use gel conductive electrodes, whereas the ECG belt employs textile embroidered electrodes. Both techniques have pros and cons. Although very comparable in terms of validity, gel electrodes tend to provide single-night ECG signals that are slightly more stable and less prone to artefacts originating from patient body position. While using the ECG belt, movement artefacts can occur more frequently and may affect R-peak detection. Filtering of the RR intervals obtained from the ECG signal from the belt is therefore particularly indicated as a preliminary step of HRV analysis [26]. On the other hand, long-term ECG monitoring using gel electrodes can trigger skin irritation and detachment. Our ECG belt is specifically adapted for long-term ECG-monitoring e.g., in a home setting [27]. The belt may, thus, be particularly useful for follow-up purpose when one needs to assess the efficacy of SAS treatment.

When using ECG signals to observe respiratory irregularities, one is in fact focusing on pathophysiological effects on other organ systems—here the cardio-circulatory system—rather than the primary respiratory effector. However, this might imply a potential asset in the sense that it is possible to not only describe a pathological phenomenon, but also shed a light on its pathogenic consequences—in particular the disturbance of the autonomous nervous system and its detrimental cardio-vascular impact. In that sense, it is interesting that we found the best discriminating power and consistent pathophysiological impact of SAS in case of an AHI >18 $h^{-1}$. Along these lines, among others, Young and colleagues [37] found that individuals with mild OSA, i.e., AHI <15 $h^{-1}$, had a similar all-cause mortality compared to individuals without OSA in contrast to individuals with an AHI of >15 $h^{-1}$. For this reason, this cut-off can be taken as a solid clinical indication for a positive airway pressure treatment.

The study is limited by the fact that we used a cross-sectional approach. In order to formally prove the usefulness and accuracy of the ECG belt for the follow-up of SAS patients, a longitudinal prospective study is needed. An algorithmic comparison was not the primary focus of the current work but was used to select an approach with appropriate performance. In the context of ECG-based apnea detection, a variety of algorithms have been described in the literature (see e.g., [19,38,39]). The current approach consisting of building a SVM classifier based on HRV-derived features is rather established. Our aim was to use a relatively straightforward algorithm in order to simplify the comparisons between ECG sensor types (patch vs. belt) and RR interval filtering (filtered vs. unfiltered). The overall performance of the SVM classifier for the classification of SAS using AHI was comparable with three alternative classifiers (Figure 2). Similarly, HRV analysis was performed using a window size of 300 s which is rather common in HRV spectral analysis. Again, in order to simplify the multiple comparisons between sensors and filters, we did not try to fine tune the window width of RR intervals. For sensitivity purpose, a shorter window size of 100 s was tested which resulted in nearly identical findings.

In future developments of the ECG belt, additional sensors such as breathing pattern detection (e.g., frequency, tidal volume), accelerometry and oxygen saturation will be included in order to further increase the diagnostic value of our wearable device. In the long run, we envisage to incorporate our technology in normal daily life textiles such as shirts (smart textiles).

## 5. Conclusions

HRV analysis from ECG signals provides a useful and accurate tool for the assessment of SAS severity in a follow-up setting—AHI range and ODI. The diagnostic accuracy obtained from filtered ECG data of the wearable ECG belt are close to the ones obtained with patched ECG during PSG. Further developments of the ECG belt in a wearable multi-channel approach should confer our wearable system a higher diagnostic accuracy of SAS.

## Figures and Tables

**Table 1:** Patient characteristics. Results are reported as median [inter-quartile range (IQR)], unless otherwise specified.

	Characteristics
**Anthropometrics**	
Subjects, *n*	241
Female/male	57/184
Age, yr	52 [IQR: 42 to 60]
BMI (kg/$m^{2}$)	45 [IQR: 27 to 61]
**Apnea severity**	
AHI, $h^{-1}$	21 [IQR: 7 to 40.2]
ODI, $h^{-1}$	17 [IQR: 4.7 to 37]
ESS, score	9 [IQR: 6 to 12]
**Type of apnea**	
Obstructive	157
Central	5
Mixed	35
No apnea detected	44

BMI: body mass index; AHI: Apnea/hypopnea index; ODI: oxygen desaturation index; ESS: Epworth sleepiness scale.

## Abbreviations

The following abbreviations are used in this manuscript:

AHI	Apnea/hypopnea index
BMI	Body mass index
ECG	Electrocardiogram
EDF	European data format
ESS	Epworth sleepiness scale
HF	High frequency
HRV	Heart rate variability
HRVi	Heart rate variability triangular index
IRRR	Inter-quartile range of the RR time series
KNN	*k*-nearest neighbour
LDA	Linear discriminant analysis
LF	Low frequency
LFHF	ratio of Low frequency on high frequency
MADRR	Median of the absolute values of the RR time series
pNN50	Proportion of interval differences of successive RR intervals greater than 50 ms
ODI	Oxygen desaturation index
OPLS	Orthogonal partial least squares
PCA	Principal Component analysis
PSG	Polysomnography
rMSSD	Root mean square of successive differences
ROC	Receiver operating characteristic
SA	Sleep apnea
SAS	Sleep apnea syndrome
SDNN	Standard deviation of the NN intervals
SVM	Support vector machine
ULF	Ultra low frequency
VLF	Very low frequency

## References

[1] Young T., Peppard P.E., Gottlieb D.J. (2002). Epidemiology of obstructive sleep apnea: A population health perspective. Am. J. Respir. Crit. Care Med..

[2] McNicholas W.T., Bonsigore M.R., Bonsignore M.R. (2007). Sleep apnoea as an independent risk factor for cardiovascular disease: Current evidence, basic mechanisms and research priorities. Eur. Respir. J..

[3] Kuhlmey F., Klotz E., Volk T., Hölzl M., Spies C., Fietze I., Dietz E., Birnbaum J. (2015). Obstructive Sleep Apnea Syndrome-Prevalence and Screening in the Preadmission Clinic. J. Anesth. Clin. Res..

[4] Young T., Peppard P., Palta M., Hla K.M., Finn L., Morgan B., Skatrud J. (1997). Population-based study of sleep-disordered breathing as a risk factor for hypertension. Arch. Intern. Med..

[5] Nieto F.J., Young T.B., Lind B.K., Shahar E., Samet J.M., Redline S., D’Agostino R.B., Newman A.B., Lebowitz M.D., Pickering T.G. (2000). Association of sleep-disordered breathing, sleep apnea, and hypertension in a large community-based study. Sleep Heart Health Study. JAMA.

[6] Garvey J.F., Pengo M.F., Drakatos P., Kent B.D. (2015). Epidemiological aspects of obstructive sleep apnea. J. Thorac. Dis..

[7] American Academy of Sleep Medicine Task Force (1999). Sleep-related breathing disorders in adults: Recommendations for syndrome definition and measurement techniques in clinical research. The Report of an American Academy of Sleep Medicine Task Force. Sleep.

[8] Flemons W.W., Douglas N.J., Kuna S.T., Rodenstein D.O., Wheatley J. (2004). Access to diagnosis and treatment of patients with suspected sleep apnea. Am. J. Respir. Crit. Care Med..

[9] Kushida C.A., Littner M.R., Morgenthaler T., Alessi C.A., Bailey D., Coleman J., Friedman L., Hirshkowitz M., Kapen S., Kramer M. (2005). Practice parameters for the indications for polysomnography and related procedures: An update for 2005. Sleep.

[10] Blackwell T., Paudel M., Redline S., Ancoli-Israel S., Stone K.L. (2017). A novel approach using actigraphy to quantify the level of disruption of sleep by in-home polysomnography: The MrOS Sleep Study: Sleep disruption by polysomnography. Sleep Med..

[11] Alshaer H., Ryan C., Fernie G.R., Bradley T.D. (2018). Reproducibility and predictors of the apnea hypopnea index across multiple nights. Sleep Sci..

[12] Collop N.A., Anderson W.M., Boehlecke B., Claman D., Goldberg R., Gottlieb D.J., Hudgel D., Sateia M., Schwab R. (2007). Clinical guidelines for the use of unattended portable monitors in the diagnosis of obstructive sleep apnea in adult patients. Portable Monitoring Task Force of the American Academy of Sleep Medicine. J. Clin. Sleep Med..

[13] Chen H., Lowe A.A., Bai Y., Hamilton P., Fleetham J.A., Almeida F.R. (2009). Evaluation of a portable recording device (ApneaLink) for case selection of obstructive sleep apnea. Sleep Breath.

[14] De Chazal P., Sadr N., Jayawardhana M. (2015). An ECG oximetry system for identifying obstructive and central apnoea events. Conf. Proc. IEEE Eng. Med. Biol. Soc..

[15] Kapoor M., Greenough G. (2015). Home Sleep Tests for Obstructive Sleep Apnea (OSA). J. Am. Board Fam. Med..

[16] Norman M.B., Middleton S., Erskine O., Middleton P.G., Wheatley J.R., Sullivan C.E. (2014). Validation of the Sonomat: A contactless monitoring system used for the diagnosis of sleep disordered breathing. Sleep.

[17] To K.W., Chan W.C., Chan T.O., Tung A., Ngai J., Ng S., Choo K.L., Hui D.S. (2009). Validation study of a portable monitoring device for identifying OSA in a symptomatic patient population. Respirology.

[18] Yadollahi A., Giannouli E., Moussavi Z. (2010). Sleep apnea monitoring and diagnosis based on pulse oximetry and tracheal sound signals. Med. Biol. Eng. Comput..

[19] Penzel T., McNames J., Murray A., de Chazal P., Moody G., Raymond B. (2002). Systematic comparison of different algorithms for apnoea detection based on electrocardiogram recordings. Med. Biol. Eng. Comput..

[20] Heima A., Karthick A., Suganthi L. Detection of sleep apnea based on HRV analysis of ECG signal. Proceedings of the International Conference on ISMAC in Computational Vision and Bio-Engineering 2018 (ISMAC-CVB).

[21] Pinho A., Pombo N., Silva B., Bousson K., Garcia N. (2019). Towards an accurate sleep apnea detection based on ECG signal: The quintessential of a wise feature selection. Appl. Soft Comput..

[22] Wang L., Lin Y., Wang J. (2019). A RR interval based automated apnea detection approach using residual network. Comput. Methods Programs Biomed..

[23] Malik M., Bigger J.T., Camm A.J., Kleiger R.E., Malliani A., Moss A.J., Schwartz P.J. (1996). Heart rate variability. Standards of measurement, physiological interpretation, and clinical use. Eur. Heart J..

[24] Lado M.J., Mendez A.J., Rodriguez-Linares L., Otero A., Vila X.A. (2012). Nocturnal evolution of heart rate variability indices in sleep apnea. Comput. Biol. Med..

[25] Weder M., Hegemann D., Amberg M., Hess M., Boesel L.F., Abacherli R., Meyer V.R., Rossi R.M. (2015). Embroidered electrode with silver/titanium coating for long-term ECG monitoring. Sensors.

[26] Fontana P., Martins N.R.A., Camenzind M., Rossi R.M., Baty F., Boesch M., Schoch O.D., Brutsche M.H., Annaheim S. (2019). Clinical Applicability of a Textile 1-Lead ECG Device for Overnight Monitoring. Sensors.

[27] Fontana P., Martins N.R.A., Camenzind M., Boesch M., Baty F., Schoch O.D., Brutsche M.H., Rossi R.M., Annaheim S. (2019). Applicability of a Textile ECG-Belt for Unattended Sleep Apnoea Monitoring in a Home Setting. Sensors.

[28] Iber C., Ancoli-Israel S., Chesson A., Quan S. (2007). The AASM Manual for the Scoring of Sleep and Associated Events: Rules, Terminology and Technical Specifications.

[29] Oksanen J. (2015). Multivariate Analysis of Ecological Communities in R: Vegan Tutorial. http://cc.oulu.fi/~jarioksa/opetus/metodi/vegantutor.pdf.

[30] R Core Team (2018). R: A Language and Environment for Statistical Computing.

[31] Rodriguez-Linares L., Mendez A.J., Lado M.J., Olivieri D.N., Vila X.A., Gomez-Conde I. (2011). An open source tool for heart rate variability spectral analysis. Comput. Methods Programs Biomed..

[32] Dray S., Dufour A. (2007). The ade4 package: Implementing the duality diagram for ecologists. J. Stat. Softw..

[33] Oksanen J., Blanchet F.G., Friendly M., Kindt R., Legendre P., McGlinn D., Minchin P.R., O’Hara R.B., Simpson G.L., Solymos P. Vegan: Community Ecology Package. https://CRAN.R-project.org/package=vegan.

[34] Gula L.J., Krahn A.D., Skanes A., Ferguson K.A., George C., Yee R., Klein G.J. (2003). Heart rate variability in obstructive sleep apnea: A prospective study and frequency domain analysis. Ann. Noninvasive Electrocardiol..

[35] Temirbekov D., Günes S., Yazıcı Z.M., Sayın İ. (2018). The Ignored Parameter in the Diagnosis of Obstructive Sleep Apnea Syndrome: The Oxygen Desaturation Index. Turk. Arch. Otorhinolaryngol..

[36] Eiseman N.A., Westover M.B., Mietus J.E., Thomas R.J., Bianchi M.T. (2012). Classification algorithms for predicting sleepiness and sleep apnea severity. J. Sleep Res..

[37] Young T., Finn L., Peppard P.E., Szklo-Coxe M., Austin D., Nieto F.J., Stubbs R., Hla K.M. (2008). Sleep disordered breathing and mortality: Eighteen-year follow-up of the Wisconsin sleep cohort. Sleep.

[38] De Chazal P., Heneghan C., Sheridan E., Reilly R., Nolan P., O’Malley M. (2003). Automated processing of the single-lead electrocardiogram for the detection of obstructive sleep apnoea. IEEE Trans. Biomed. Eng..

[39] Lado M.J., Vila X.A., Rodriguez-Linares L., Mendez A.J., Olivieri D.N., Felix P. (2011). Detecting sleep apnea by heart rate variability analysis: Assessing the validity of databases and algorithms. J. Med. Syst..

